# Microbial Communities in Methane- and Short Chain Alkane-Rich Hydrothermal Sediments of Guaymas Basin

**DOI:** 10.3389/fmicb.2016.00017

**Published:** 2016-01-29

**Authors:** Frederick Dowell, Zena Cardman, Srishti Dasarathy, Matthias Y. Kellermann, Julius S. Lipp, S. Emil Ruff, Jennifer F. Biddle, Luke J. McKay, Barbara J. MacGregor, Karen G. Lloyd, Daniel B. Albert, Howard Mendlovitz, Kai-Uwe Hinrichs, Andreas Teske

**Affiliations:** ^1^Department of Marine Sciences, University of North Carolina at Chapel HillChapel Hill, NC, USA; ^2^MARUM Center for Marine Environmental Sciences and Department of Geosciences, University of BremenBremen, Germany; ^3^Department of Earth Science and Marine Science Institute, University of California at Santa BarbaraSanta Barbara, CA, USA; ^4^HGF-MPG Group for Deep-Sea Ecology and Technology, Max Planck Institute for Marine MicrobiologyBremen, Germany; ^5^School of Marine Science and Policy, University of DelawareLewes, DE, USA; ^6^Department of Microbiology, The University of TennesseeKnoxville, TN, USA

**Keywords:** Guaymas Basin, ANME, alkanes, methane, sediment, hydrothermal vents

## Abstract

The hydrothermal sediments of Guaymas Basin, an active spreading center in the Gulf of California (Mexico), are rich in porewater methane, short-chain alkanes, sulfate and sulfide, and provide a model system to explore habitat preferences of microorganisms, including sulfate-dependent, methane- and short chain alkane-oxidizing microbial communities. In this study, hot sediments (above 60°C) covered with sulfur-oxidizing microbial mats surrounding a hydrothermal mound (termed “Mat Mound”) were characterized by porewater geochemistry of methane, C_2_–C_6_ short-chain alkanes, sulfate, sulfide, sulfate reduction rate measurements, *in situ* temperature gradients, bacterial and archaeal 16S rRNA gene clone libraries and V6 tag pyrosequencing. The most abundantly detected groups in the Mat mound sediments include anaerobic methane-oxidizing archaea of the ANME-1 lineage and its sister clade ANME-1Guaymas, the uncultured bacterial groups SEEP-SRB2 within the *Deltaproteobacteria* and the separately branching HotSeep-1 Group; these uncultured bacteria are candidates for sulfate-reducing alkane oxidation and for sulfate-reducing syntrophy with ANME archaea. The archaeal dataset indicates distinct habitat preferences for ANME-1, ANME-1-Guaymas, and ANME-2 archaea in Guaymas Basin hydrothermal sediments. The bacterial groups SEEP-SRB2 and HotSeep-1 co-occur with ANME-1 and ANME-1Guaymas in hydrothermally active sediments underneath microbial mats in Guaymas Basin. We propose the working hypothesis that this mixed bacterial and archaeal community catalyzes the oxidation of both methane and short-chain alkanes, and constitutes a microbial community signature that is characteristic for hydrothermal and/or cold seep sediments containing both substrates.

## Introduction

The Guaymas Basin in the Gulf of California is a young marginal rift basin characterized by active seafloor spreading and rapid deposition of organic-rich sediments derived from both, highly productive overlying waters as well as terrigenous input. Within Guaymas Basin sediments, deeply emplaced volcanic dikes and sills originating from the spreading center have indurated and hydrothermally altered their surrounding sediment matrix, and continue to shape hydrothermal circulation patterns today (Einsele et al., 1980; Lonsdale and Becker, 1985). Hydrothermal pyrolysis at increased temperature and pressure transforms and mobilizes a major proportion of buried organic matter thereby generating complex petroleum-like compounds (Simoneit and Lonsdale, 1982; Kawka and Simoneit, 1987; Bazylinski et al., 1988; Didyk and Simoneit, 1989). Furthermore, low-molecular weight organic acids (Martens, 1990) and ammonia (Von Damm et al., 1985) are produced in large amounts. As these substrates are entrained into hydrothermal circulation and reach cooler overlying sediments, they are assimilated by dense microbial communities in the surficial sediments (Pearson et al., 2005). The benthic microbial communities of Guaymas Basin mediate methanogenesis (Jones et al., 1983), methane oxidation (Kallmeyer and Boetius, 2004), and sulfate reduction (Jørgensen et al., 1990, 1992; Elsgaard et al., 1994; Weber and Jørgensen, 2002; Kallmeyer et al., 2003), among other processes. This active microbial interface, also termed “microbial gauntlet” (Teske et al., 2014), could modulate hydrothermal carbon mobilization and release into the environment (Lizarralde et al., 2011).

The hydrothermal sediments of Guaymas Basin support a microbial process found throughout marine sediments: sulfate-dependent anaerobic oxidation of methane (AOM) by multiple types of anaerobic methanotrophic archaea (ANME-1, ANME-2, and ANME-3), acting in syntrophic partnership with specific sulfate-reducing bacteria that form morphologically complex consortia with ANME archaea (Knittel and Boetius, 2009). The electrons that are transferred from methane-oxidizing archaea (ANME-2) to their sulfate-reducing partners are not exchanged by diffusion of a reduced substrate, but appear to be transferred directly by ANME multi-haem cytochromes; these are embedded in the cellular membranes of both partners and also occur in the interstitial space between the cells (McGlynn et al., 2015).

Initial evidence for AOM in Guaymas Basin sediments came from 16S rRNA gene sequences of ANME archaea and compound-specific δ^13^C-signatures of archaeal lipids (Teske et al., 2002). Further evidence came from activity measurements in *ex situ* laboratory incubations at high temperatures (Kallmeyer and Boetius, 2004; Holler et al., 2011; Kellermann et al., 2012), and FISH hybridization of ANME archaea, in part associated with bacterial syntrophs in high-temperature enrichments (Holler et al., 2011). These thermophilic sulfate-reducing syntrophs – the HotSeep-1 bacteria - are distinct from the sulfate-reducing syntrophs that dominate in mesophilic or cold-seep consortia; they can grow independently as hydrogenotrophs but form cell-to-cell conduits for direct electron exchange with their ANME-1 partners (Wegener et al., 2015). Phylotypes of the recently identified lineages ANME-1a Guaymas I and II (specific clusters within ANME-1; Holler et al., 2011) and of the ANME-1 sister lineage ANME-1Guaymas (Biddle et al., 2012) were recovered in both hot and cold sediments of Guaymas Basin, implying tolerance of a wide *in situ* temperature range among these groups.

The evidence for anaerobic short-chain alkane oxidation in Guaymas Basin under mesophilic and thermophilic conditions is based to a large extent on pure culture studies. Mesophilic sulfate-reducing bacteria that oxidize *n*-butane (Kniemeyer et al., 2007; Jaekel et al., 2013), thermophilic sulfate-reducing enriched cultures oxidizing *n*-butane and propane (Kniemeyer et al., 2007), and thermophilic sulfate-reducing bacterial isolates oxidizing *n*-decane (Rüter et al., 1994) have been obtained from Guaymas Basin hydrothermal sediments. A prominent uncultured group within the *Deltaproteobacteria*, the SEEP-SRB2 lineage, appears conspicuously – sometimes as ANME syntrophs, sometimes as free-living cells – in methane- and short chain alkane-rich seep sediments, including Guaymas Basin; SEEP-SRB2 has been proposed as a sulfate-reducing ethane oxidizer (Kleindienst et al., 2012).

In this study, we extend the survey of different Guaymas Basin locations to include the hot mat-covered sediments surrounding a large mound of hydrothermal seafloor deposits (Peter and Scott, 1988), and we develop working hypotheses for the environmental controls that foster different ANME lineages and candidate lineages for sulfate-reducing short-chain alkane oxidation in Guaymas Basin sediments and beyond.

## Materials and Methods

### Sediment Sampling and *In Situ* Temperature Profiling

**FIGURE 1:**
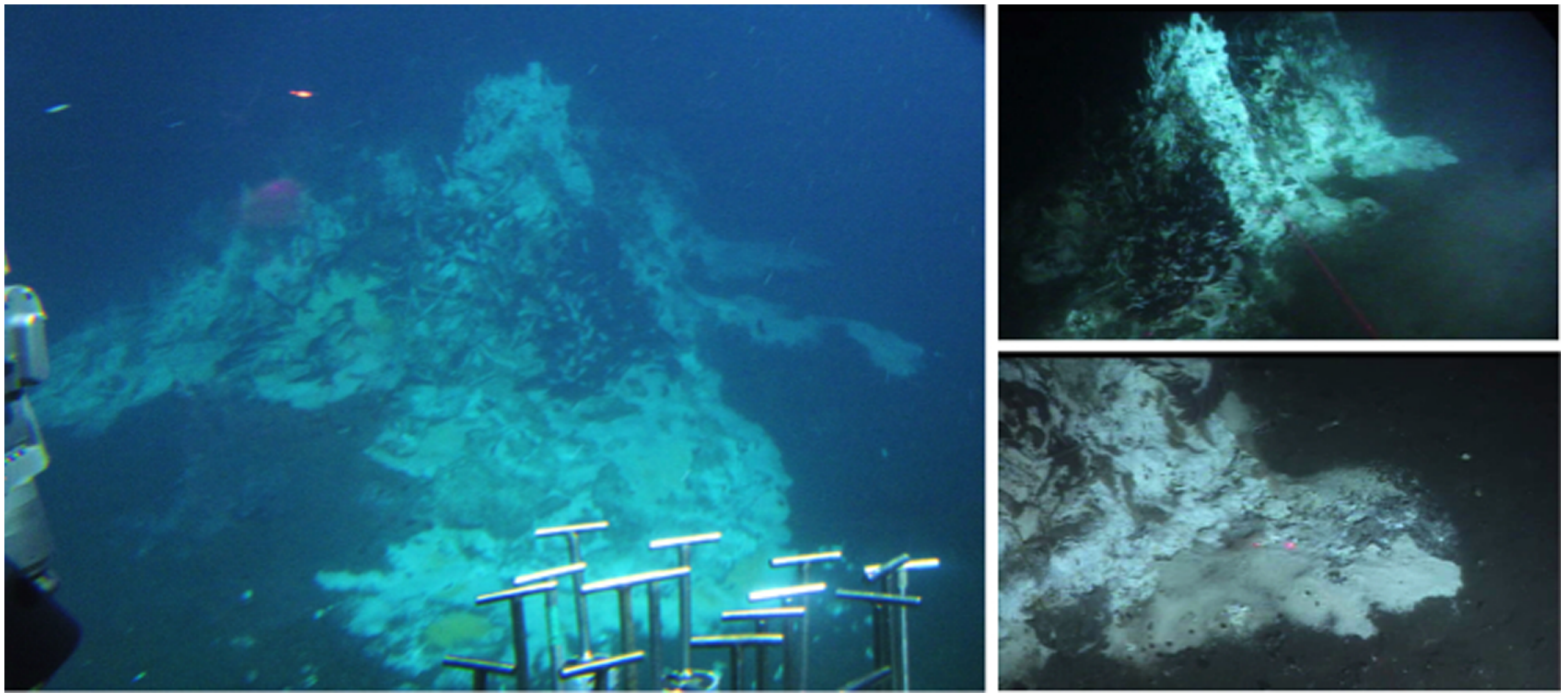
**(**Left** and **Top Right**) Panoramic views of Mat Mound, discovered during *Alvin* dive 4483**. The mound is ca. 2.5 m high and several meters across. **(Bottom Right)** The basis of Mat Mound with close-up of the principal microbial mat area to be cored, photographed during dive 4484 before cores were taken. The two red laser points mark a distance of 10 cm. Small venting holes in the mat are visible in the mat foreground.

With the deep-sea submersible *Alvin*, thermal gradients were measured and push cores were collected from hydrothermally active sediments at the base of Mat Mound. Mat Mound is a small mound of hydrothermal precipitates, approximately two and a half meters high from the base to the top (**Figure 1**) and is located in the southern rift valley of the Guaymas Basin (27°N00.388, 11°W24.560). During *Alvin* dive 4484 (December 7th, 2008), the temperature profiles and sediment cores used in this study were obtained from a specific hydrothermal mat area, ca. 70 cm across, at the base of Mat Mound (Supplementary Figures S1 and S2). Prior to coring, temperature profiles at the sampling site were recorded using *Alvin*’s external heatflow temperature probe, a 0.6 m titanium tube containing a linear heater and five thermistors (type 44032, Omega Engineering, Inc.) at 10 cm intervals along the length of the tube (McKay et al., 2012). When fully immersed in the sediment, this probe records five *in situ* temperatures at the sediment/water interface, and at 10, 20, 30, and 40 cm sediment depth. The closest available temperature profile, 4484-M4-HF3 in the center of the mat, was used to estimate the thermal regime in nearby core 4484-1. After completing the temperature gradient survey, sediment push cores were collected using 45 cm long polycarbonate cores with a 6.25 cm interior diameter. All cores were returned to the ship within 2–4 h of collection. After 4–10 h of temporary shipboard storage at 4°C, the geochemistry core was subsampled in 2 cm intervals, and the core dedicated to molecular biological studies was subsampled in 1 cm intervals, in both cases at room temperature. The molecular biology samples were stored at –80°C on the ship, transported on dry ice to the home laboratory at the University of North Carolina at Chapel Hill, NC, USA, and stored at –80°C until further processing.

### DNA Extraction and PCR

Microbial DNA was extracted from 0.5 g of sediment with a PowerSoil DNA Kit (MoBio Laboratories, Inc., Carlsbad, CA, USA) for PCR amplification. The bacterial and archaeal 16S rRNA genes were amplified with primer combinations B8f (5′-AGRGTTTGATCCTGGCTCAG-3′) and B1492r (5′-CGGCTACCTTGTTACGACTT-3′), and A8f (5′-TCCGGTTGATCCTGCC-3′) and A1492r (5′-GGCTACCTTGTTACGACTT-3′), respectively (Teske et al., 2002). When full-length archaeal PCR amplicons could not be obtained in the 0-1 cm depth interval, the A1492r primer was substituted with A915r (5′-GTGCTCCCCCGCCAATTCCT-3′; Stahl and Amann, 1991), resulting in a shorter 16S rRNA gene PCR amplicon.

The PCR products for both archaeal and bacterial samples were gel purified with the Promega Wizard SV Gel and PCR-Clean-Up System (Promega Corp., Madison, WI, USA) following the manufacturer’s instructions in order to remove contaminants in the sample that could have interfered with cloning. The purified products were ligated into PCR 2.1 TOPO Cloning Vector plasmid containing β-galactosidase and kanamycin resistance genes. Competent TOP-10 *Escherichia coli* cells were transformed using the TOPO TA Cloning Kit (Invitrogen, Inc., Carlsbad, CA, USA), and then plated onto minimal media agar plates containing bromo-chloro-indolyl-galactopyranoside (X-GAL) and kanamycin for blue/white screening. After 24 h of incubation at 37°C, white colonies were picked. The colonies were re-plated after another 24 h for Sanger sequencing (GENEWIZ, Inc., South Plainfield, NJ, USA) using M13 (–20) forward and M13 (–20) reverse primers. As a precaution, copies of the colonies were placed in glycerol stocks (85% S.O.C. medium and 15% glycerol) and stored at –80°C for resequencing.

### DNA Sequencing and Phylogenetic Analysis

Sequence contigs were constructed and analyzed for read quality using Sequencher software (Genecodes, Inc., Ann Arbor, MI, USA). Ambiguous bases were corrected according to the complementary base pair when possible. Chimeras were identified with Pintail 1.0 (Ashelford et al., 2005), and removed. Full sequences (combining from forward and reverse reads) were analyzed for initial identification using BLASTN^1^. Aligned sequences were phylogenetically assigned based on their placement within distance-based neighbor-joining phylogenies in the ARB software platform^2^. The branching pattern of these phylogenies was assessed with 1,000 bootstrap reiterations in ARB (Ludwig et al., 2004). Sequences are deposited in GenBank under accession numbers KJ569632 to KJ569688.

### Pyrosequencing of 16S rRNA Gene Fragments

The hypervariable V6 region of the 16S rRNA gene was PCR amplified using one forward and two reverse primers for archaea (Arch958F/Arch1048Rmix) and four forward and four reverse primers for bacteria (Bac967Fmix/ Bac1064Rmix). Primer details are provided on the website “Visualization and Analysis of Microbial Population Structures” (VAMPS) of the Marine Biological Laboratory, Woods Hole^3^. Massively parallel tag sequencing of the PCR products was carried out on a 454 Life Sciences GS FLX sequencer at Marine Biological Laboratory, Woods Hole, MA, USA. The sequences were submitted to a rigorous quality control procedure based on mothur v24 (Schloss et al., 2009), which includes denoising of the flow grams using an algorithm based on PyroNoise (Quince et al., 2009), single-linkage preclustering (Huse et al., 2010) and removal of PCR errors and a chimera check using Uchime (Edgar et al., 2011). Archaeal and bacterial sequences longer than 79 and 74 bases, respectively, were clustered at 97% sequence identity (OTU_0.03_) and taxonomically assigned based on SILVA as implemented in mothur. For archaea we used release 102, 02-2010; for bacteria release 119, 07-2014 (Quast et al., 2013). To minimize biases all steps were performed according to the same protocols using the same infrastructure.

### Geochemical Analyses

Porewater geochemistry was analyzed as described previously (Biddle et al., 2012). Sulfate concentration measurements were completed shipboard; after centrifuging sediment-filled 15 ml tubes, the overlying porewater was filtered through 0.45 μm filters, acidified with 50 μl of 50% HCl and bubbled with nitrogen for 4 min to remove sulfide. Sulfate concentrations were then measured shipboard using a 2010i Dionex Ion Chromatograph (Sunnyvale, CA, USA) through Ag^+^ exchange columns (Dionex) so as to remove Cl^-^ (Martens et al., 1999). For sulfide, 1 ml porewater samples were combined with 0.1 M zinc acetate and concentrations were analyzed spectrophotometrically on the ship (Cline, 1969). Headspace methane concentrations for core 4484-3 were determined onboard by standard gas chromatography with a flame ionization detector (FID), specifically using a HACH Carle Series 100 AGC Gas Chromatograph with a Alltech Molecular Sieve 5 A packed column (80/100 mesh, 3.05 m length, 3.2 mm ID) and a 80°C isothermal temperature profile. Stable isotopic compositions of the same methane samples (core 4484-3) were measured post-cruise at UNC via gas chromatography-combustion-isotope ratio mass spectrometry (GC-C-IRMS) on a Finnigan MAT 252 Isotope Ratio Mass Spectrometer, using a HP 5890 Series II Gas Chromatograph with a HP Plot Q column (30 m length, 0.32 mm ID, 20 μm film thickness) and a 30°C isothermal temperature profile. To measure DIC, 2 ml of unamended porewater from each sediment horizon were injected into evacuated serum vials (30 ml) and stored upside down at –20°C. At UNC, the samples were thawed, and DIC was reacted to gaseous CO_2_ by adding 1 ml of a 30% phosphoric acid solution to each serum vial and shaking vigorously before GC analysis (Kelley et al., 1990). Stable isotopic values and concentrations of DIC were analyzed via coupled GC (Hewlett Packard, 5890) and Isotope Ratio Mass Spectrometer (Finnigan MAT 252).

For combined analysis of methane and its higher gaseous homologs from core 4484-6 and 4484-16, sediment subsamples were added to serum bottles containing a 1 M sodium hydroxide solution. Methane, ethane, propane, and short-chain *n*-alkanes up to hexane (C_1_ to C_6_) in cores 4484-6 and 4484-14 were identified either by comparing the retention time of authentic standards (100 ppm C1–C6 standards, Sigma–Aldrich Chemie GmbH, Munich, Germany) or by gas chromatography–mass spectrometry (GC–MS) analysis of selected headspace samples, as described previously (Xie et al., 2013). For quantification, an aliquot (100 μl) of gas was then taken from the headspace using a Hamilton SampleLock syringe for on-column injection via a programmable temperature vaporizing inlet. For GC quantitation, we used a Trace GC Ultra (ThermoFinnigan GmbH, Bremen, Germany) equipped with a CP-PoraBOND Q column (Agilent Technologies Deutschland GmbH, Böblingen, Germany) and FID. The column temperature program was as follows: 60°C (1 min) to 240°C (held 2 min) at a rate of 40°C min^-1^. For calibration, different quantities of a 100 and 1000 ppm C_1_ to C_6_ standard were injected, ranging from 10 to 400 ppm to reassure linear response of the FID. A potential isotope effect of alkane transition from dissolved to gaseous phase was checked with measurements of known standards in a comparable sampling setup; no obvious isotope effect was found. For stable carbon isotope analysis, the same model of gas chromatograph was used and coupled to a Delta Plus XP isotope ratio mass spectrometer via a combustion interface-III (all from ThermoFinnigan GmbH). A column and a temperature program identical to those described above were used. The internal precision was better than ±0.1‰ (1 SD).

Hydrogen measurements followed a previously published protocol (Lin et al., 2012). A sediment subsample of 2–3 mL was extruded into a 12 mL headspace vial right after core retrieval, quickly sealed with a thick pre-cleaned butyl rubber stopper (Glasgerätebau Ochs GmbH, Bovenden, Germany). The vial was crimp-capped, and flushed with ultrapure N_2_ for longer than 1 min. The collected subsamples were incubated in the dark close to *in situ* temperature, and H_2_ concentrations in the headspace gas were analyzed daily, aiming to reach steady-state conditions. All shipboard incubations were conducted as long as possible, up to 8 days and a minimum of 3 days toward the end of the cruise. In order to compensate for decreasing gas pressure in the headspace due to sampling, sample volumes were immediately replaced with an equal amount of ultrapure N_2_ (1 mL), which was injected into the headspace. The H_2_ headspace gas concentration was analyzed using a Peak Performer 1 gas chromatograph (Peak Laboratories, LLC, USA). All gas samples and gas standards were injected into the GC using a gas-tight syringe; with ultrapure N_2_ gas as carrier, the gas sample was then separated on a packed column before ultimately reaching the mercuric oxide detector.

Sulfate reduction rates were determined in shipboard *ex situ* incubations at room temperature (near 20°C) using 2 cm sediment layers of cores 4484-3 and 4484-10, following previously published protocols for marine sediments (Hoehler et al., 1994).

## Results and Discussion

### Thermal and Biogeochemical Habitat Characterization

**FIGURE 2:**
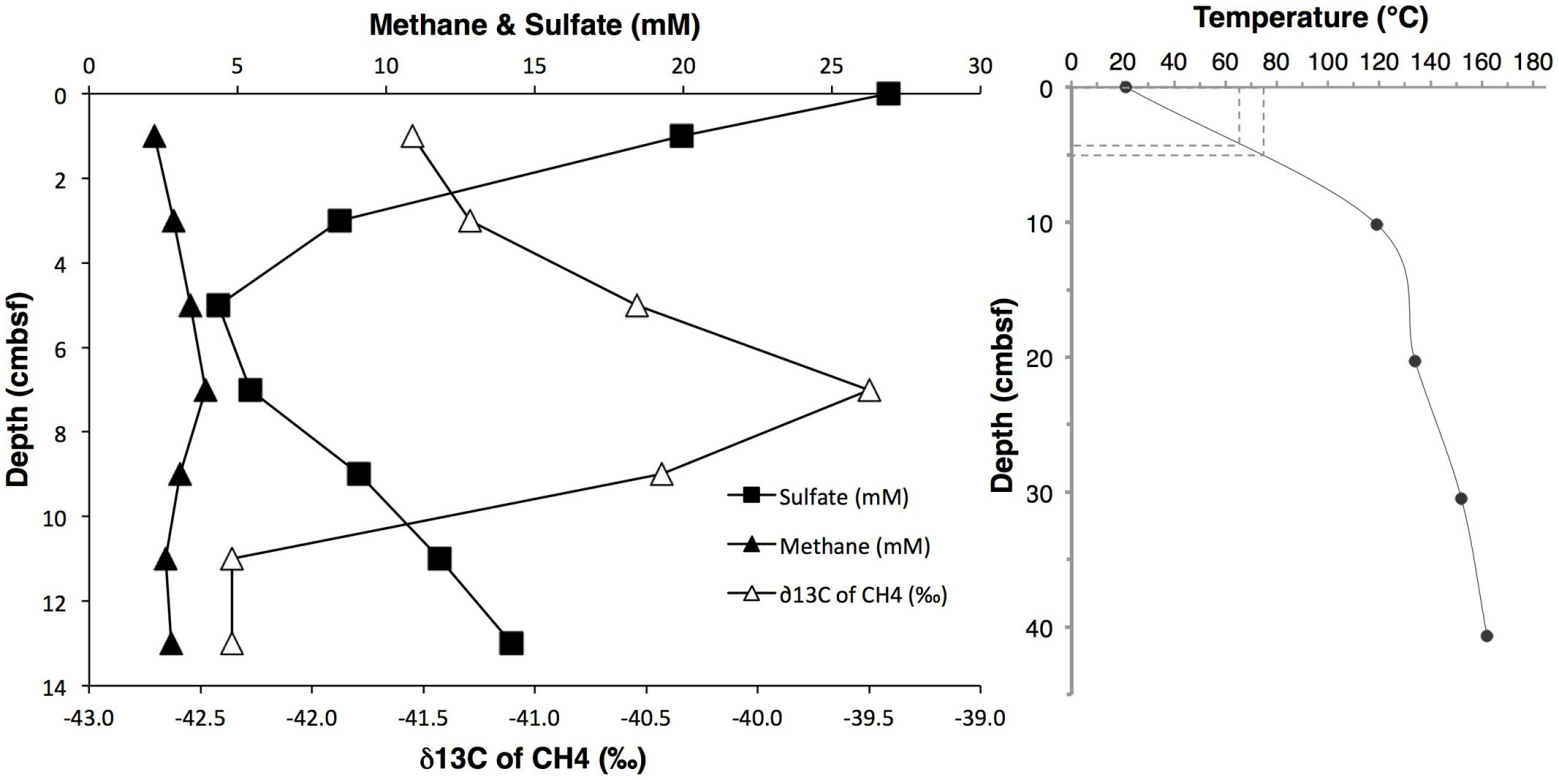
**(Left)** Profiles of dissolved methane concentrations, δ^13^C-CH_4_ isotopic signatures, and sulfate concentrations, obtained from core 4484-3. δ^13^C-CH_4_ values span a narrow range of approximately 3‰, and are plotted at high δ resolution. **(Right)** *In situ* temperature gradient obtained by Heatflow Probe measurement #3 (see Supplementary Figure S2C for location). The hyphenated lines indicate the 4–5 cm sample interval and its interpolated temperature range.

The investigated sediment samples were located at the base of a small hydrothermal mound termed “Mat Mound,” overgrown with *Riftia* and microbial mats of white sulfur bacteria which indicated diffuse venting of sulfidic fluids through the porous matrix of the mound (**Figure 1**). Extensive *in situ* temperature profile measurements made during Alvin dives 4483, 4484, 4485, and 4488 showed steep hydrothermal gradients within the sediments at the base of the mound, indicating that the hydrothermal heat and fluid transport is actively channeled along the interface between the mound and surrounding sediment, thus supporting a fringe of abundant microbial mats (Supplementary Figure S2C). A drop in temperature in the sediment within a short distance corresponds with a decrease in hydrothermal fluid supply and is accompanied by the disappearance of microbial mats (McKay et al., 2012). The steepest temperature profile HF3 (**Figure 2**, and Supplementary Figure S2) rose rapidly from 20°C at the seawater/sediment interface to approximately 119°C at a depth of about 10 cm bsf, and to 162°C at a depth of 40 cm bsf. Subsamples from the adjacent sediment core 4484-1 were used for DNA extraction and sequencing, and encompassed a steep temperature gradient ranging from approximately 20°C at 0–1 cm sediment depth toward the interpolated range of approximately 65 to 75°C at 4–5 cm depth (**Figure 2**). The nearest geochemistry profile, core 4484-3, was located approximately 30 cm from the measured temperature profile and the DNA samples (Supplementary Figure S2C) and showed methane concentrations of ca. 2.2 to 3.4 mM within the upper 5 cm (**Figure 2** and Supplementary Table S1). Due to methane outgassing during core retrieval, these shipboard measurements most likely underestimated the *in situ* methane concentrations; solving this problem requires gas-tight sample collection under pressurized conditions (Seewald et al., 2001). The complex profile of δ^13^C-CH_4_ in core 4484-3 most likely reflects influences of microbial methane production and oxidation and of advective mixing, but it remains within the relatively narrow range of –42.4 to –39.5‰ (**Figure 2**). This range is close to the values reported previously for hydrothermal methane in Guaymas Basin, a mixture originating from thermal degradation of buried photosynthetic biomass in the sediments, and from hydrothermal fluids (Welhan, 1988; Pearson et al., 2005). These results suggest that microbial methanogenesis and methane oxidation impact the overall isotopic signature of the porewater methane pool in core 4484-3 only to a moderate extent. In contrast, the lower porewater methane concentrations (1.6 to 1.8 mM) and higher δ^13^C-CH_4_ (–28.0 to –20.6‰; Supplementary Table S1) in the upper 5 cm of the cooler core 4483-23, located on the periphery of the Mat Mound hydrothermal sediments (Supplementary Figure S1), indicate a stronger microbial methane oxidation imprint on the smaller methane pool in this cooler sediment.

Porewater sulfate decreased from near seawater concentration (28 mM) at 0–1 cm depth toward a local minimum concentration of approximately 4.3 mM at 4–5 cm depth in core 4484-3, before increasing again at greater depth (**Figure 2**), presumably due to inmixing of seawater entrained laterally by hydrothermal circulation. The conspicuous near-surface sulfate concentration decrease was consistent with sulfate reduction activity in the surficial sediments: Mesophilic sulfate reduction rates measured *ex situ* in core 4484-3 at 20°C dropped by an order of magnitude from >300 nmol ml^-1^ day^-1^ at the sediment surface (0–2 cm) to approximately 30 nmol ml^-1^ day^-1^ at 2–4 cm depth, and to approximately 10 nmol ml^-1^ day^-1^ to 4–6 cm depth (Supplementary Figure S3). Mesophilic sulfate reduction activities in core 4484-10 (located near the heatflow profile 4 toward the margin of the mat; Supplementary Figure S2C) extended deeper into the sediment and remained detectable at 6 to 8 cm depth (Supplementary Figure S3). Sulfide concentrations were not determined in core 4484-3, but sulfide profiles in other cores obtained from mat-covered sediments around Mat Mound revealed consistent porewater sulfide maxima in the range of 1–3 mM at 5–10 cm depth (Supplementary Table S2).

Porewater DIC concentrations and δ^13^C signatures, as far as determined for cores 4483-23 and 24 in Mat Mound sediments, ranged from 4.7 to 17.3 mM and –7.0 to –23.1‰, respectively (Supplementary Table S1), and were consistent with mixed sources of volcanic origin and remineralization of organic matter (Pearson et al., 2005).

**FIGURE 3:**
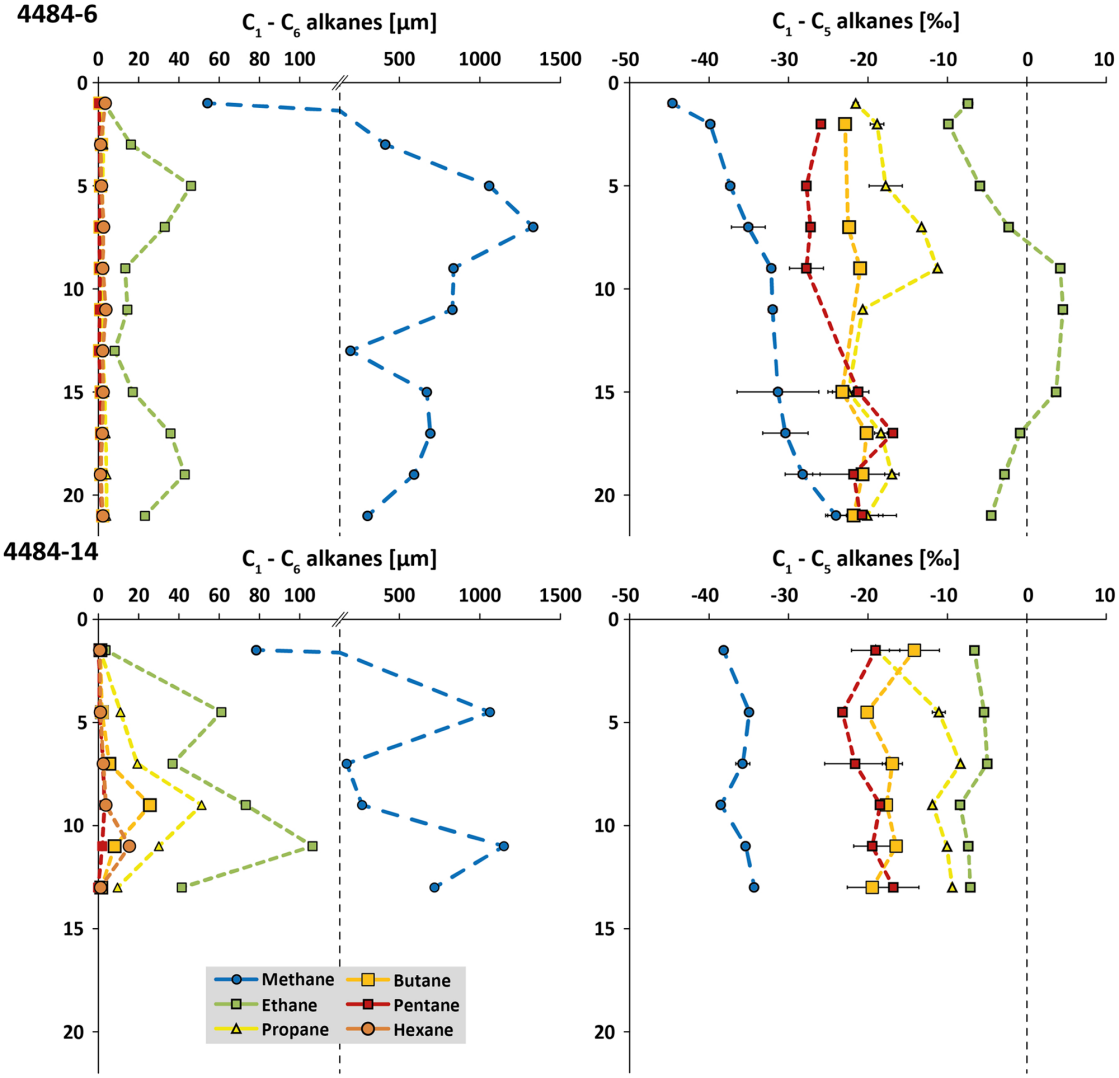
**Concentration profiles (in μM) and δ^13^C values (in **‰**) for short-chain alkanes (C_1_ to C_6_) from Guaymas sediment cores 4484-6 and 4484-14**. The concentration scale is discontinuous to accommodate methane as well as the short-chain alkanes within the same plots. When available, error bars from triplicate measurements are included.

Two sediment cores from opposite sides of the same mat-covered sampling area, cores 4484-6 and 4484-14 (Supplementary Figure S2C), were analyzed for concentrations and δ^13^C of short-chain alkanes, from methane to hexane (**Figure 3**). Similar to previous observations (Biddle et al., 2012), porewater methane reached millimolar concentrations in some sediment horizons and ranged in δ^13^C from –25 to –45‰; most data points fell between –30 to –40‰. The high variability of the methane concentration profile, and the unusual trends of increasing ^13^C depletion in methane toward the surface, suggest a complex overlay of different advection regimes around the hydrothermal mound, and of biogenic isotopic imprints such as methane oxidation within the deeper sediment layers and/or methanogenesis in the shallower layers of the core. Ethane concentrations reached 40–100 μM in some sediment horizons, where its maxima were congruent with those of methane. Ethane is considerably ^13^C-enriched and shows δ^13^C signatures mostly in the range from –10 to 0‰, even approaching positive values of up to +5‰ in three consecutive horizons of core 4484-6 (**Figure 3**). These heavy ethane signatures contrast with probably biogenic ^13^C-depleted ethane (–44‰ to –70‰) that have been reported previously in anoxic marine sediments (Waseda and Didyk, 1995; Seifert et al., 1999; Paull et al., 2000). A thermogenic source would be consistent with higher C_2_/C_1_ ratios in hydrothermally influenced subsurface sediments of Guaymas Basin (Simoneit and Philp, 1982; Whelan et al., 1988). It should also be noted that the trend of increasing ^13^C depletion in gaseous alkanes of increasing carbon number, from ethane toward *n*-pentane, resembles trends observed for C_1_ to C_4_ abiogenic hydrocarbons detected in crystalline rocks of the Canadian Shield (Sherwood Lollar et al., 2002), although the organic-rich Guaymas sediments represent an obviously different geological setting. Propane, *n*-butane, *n*-pentane, and *n*-hexane concentrations remained below those of ethane, either in the low micromolar range or below detection limit; the δ^13^C signatures of the samples with measurable concentrations are intermediate between methane and ethane (**Figure 3**). Consistent with higher short-chain alkane concentrations in core 4484-14, this core was located closer to the hot, hydrothermally active base of Mat Mound than the peripherally located core 4484-6 (Supplementary Figure S2C).

**FIGURE 4:**
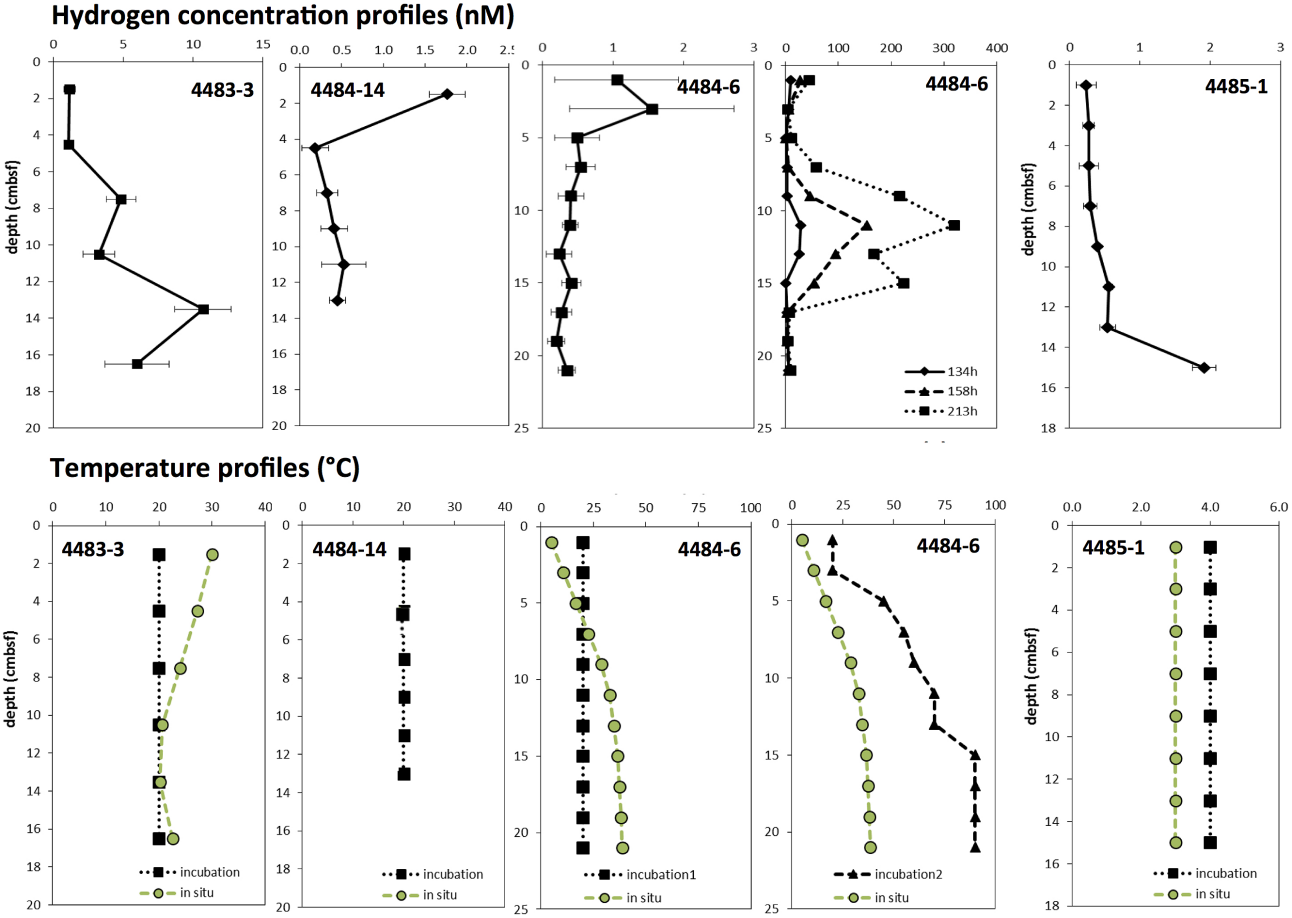
**Hydrogen concentrations, shown as averages with standard deviations, after >72 h of pre-incubation at 20°C for all core depths (two **Left** panels, and **Center** panel), at increasing temperatures downcore (second panel from the **Right**), and at 4°C for all core depths **(Right)****. The warm cores 4483-3, 4484-14, and 4484-6 were obtained from the mat-covered periphery of Mat Mound; the cool background core 4485-1 represents bare sediment at approximately 1 m distance from Mat Mound. Prolonged incubation (134–213 h) of core 4484-6 at >50°C shown in the second panel from the right resulted in localized H_2_ accumulation in the range of 100 to 300 nM, indicating that microbial H_2_ production can exceed consumption under these conditions. Interpolated temperatures for every incubated sediment sample are shown in the temperature profiles below each hydrogen profile; they are based on the *in situ* positions of cores and temperature profiles in Supplementary Figure S1 (core 4483-3, profile HF7; and core 4485-1, profile HF1) and Supplementary Figure S2C (core 4484-6, profile HT2). Core 4484-14 has no nearby temperature profile, but its position near the base of Mat Mound suggests high temperatures.

Porewater hydrogen concentrations profiles in Mat Mound cores peaked between 2 to 10 nM after pre-incubation at mesophilic temperatures (**Figure 4**). The hydrogen concentrations were consistent with published hydrogen concentrations in anaerobic sediments (Lin et al., 2012). Specifically, the concentration range of extracted hydrogen was compatible with microbial consumption and cycling of hydrogen in sulfate-reducing marine sediments, but remained too low to sustain hydrogenotrophic methanogenesis (Lin et al., 2012). As shown for the Mat Mound sediments (**Figure 2**, and Biddle et al., 2012) and many other hydrothermal hot spots in Guaymas Basin, surficial hydrothermal sediments are generally permeated with porewater sulfate (Jørgensen et al., 1990; Weber and Jørgensen, 2002; Biddle et al., 2012), almost certainly due to seawater entrainment by hydrothermal pumping (Gundersen et al., 1992; Winkel et al., 2014). The widely available microbial electron acceptor sulfate is suitable for selectively scavenging hydrogen from diffusely venting hydrothermal fluids (Wankel et al., 2011). In consequence, methanogenic communities in Guaymas Basin include genera that do not require hydrogen, but specialize in non-competitive substrates that are not used by sulfate-reducing bacteria (Lever and Teske, 2015). As a caveat against overgeneralization of this argument, higher *in situ* temperatures or incubation temperatures could lead to increased hydrogen concentrations (**Figure 4**), and may therefore affect hydrogen-dependent microbial processes and populations.

### Microbial Community Analyses

The depth intervals from 0 to 1 and 4 to 5 cm of core 4484-1 were chosen for sequence-based microbial community analyses (nearly full-length 16S rRNA gene clone libraries and V6 pyrosequencing) based on the *in situ* temperature regime, and nearby porewater gradients of sulfate, methane, and δ^13^C of methane from core 4484-3, which indicated a sulfate-and methane-rich environment suitable for anaerobic methane oxidation at moderate to high temperatures.

**FIGURE 5:**
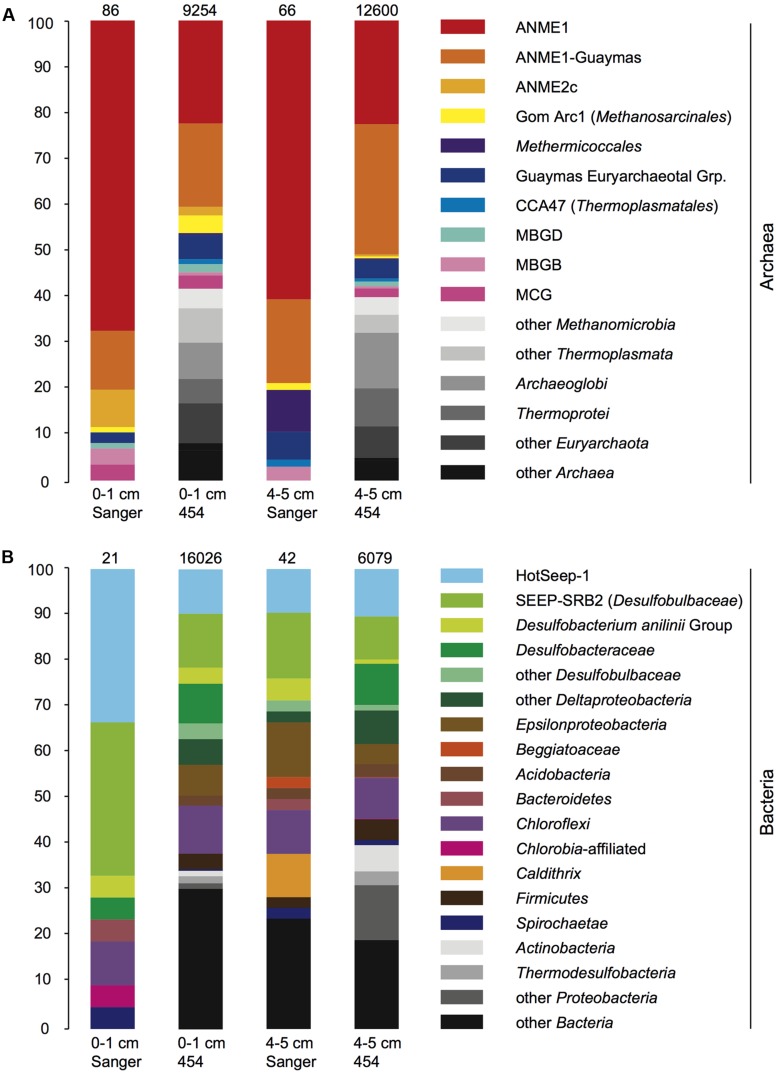
**(A,B)** Bar diagrams of bacterial and archaeal clone library and V6-tag composition, for DNA-based PCR amplicons in the 0–1 and 4–5 cm sediment layers. The phylogenetic groups that are found only in the V6-tag survey but not in the clone libraries appear in different shades of gray.

The V6-tag sequencing results shared key features with the 16S rRNA gene clone library sequencing surveys, including the abundant detection of the ANME-1 archaea in the archaeal community. Both the 16S rRNA gene and V6-tag analyses showed that ANME-1 and ANME-1Guaymas constitute the two most frequently found archaeal groups; together they accounted for four fifths of the archaeal clones and nearly half of the archaeal V6-tag dataset (**Figure 5A**). The archaeal clone library and V6-tag dataset of the surface sediment, but not the deeper sediment layer, also include ANME-2c sequences. Interestingly, the archaeal V6-tag data contain additional taxa that are not found in the clone libraries (marked in gray shades in **Figure 5**); these differences may represent greater efficiency of detecting rare taxa by high-throughout sequencing, or compositional differences in the pools of intact DNA and fragmented DNA that were accessible by full length 16S rRNA gene amplification, cloning, and sequencing, compared to pyrosequencing of partial (V6) 16S rRNA gene amplicons. The two most conspicuous bacterial groups, HotSeep-1 and the deltaproteobacterial lineage SEEP-SRB2, accounted together for two thirds of the 16S rRNA gene clones of the 0–1 cm layer or for approximately one fifth in the 16S rRNA gene clones of the 4–5 cm layer, and for the same proportion in both V6-tag datasets (**Figure 5**). Diverse *Deltaproteobacteria*, *Epsilonbacteria*, *Chloroflexi*, and other bacterial groups constituted most of the remaining bacterial community components. The bacterial and archaeal V6-tag surveys detected members of hyperthermophilic sulfate-reducing lineages, such as *Archaeoglobus* and *Thermodesulfobacteria*; these plausible inhabitants of hydrothermal sediments appear to have been overlooked by the clone libraries.

### Diversity and Function of Major Archaeal Groups

**FIGURE 6:**
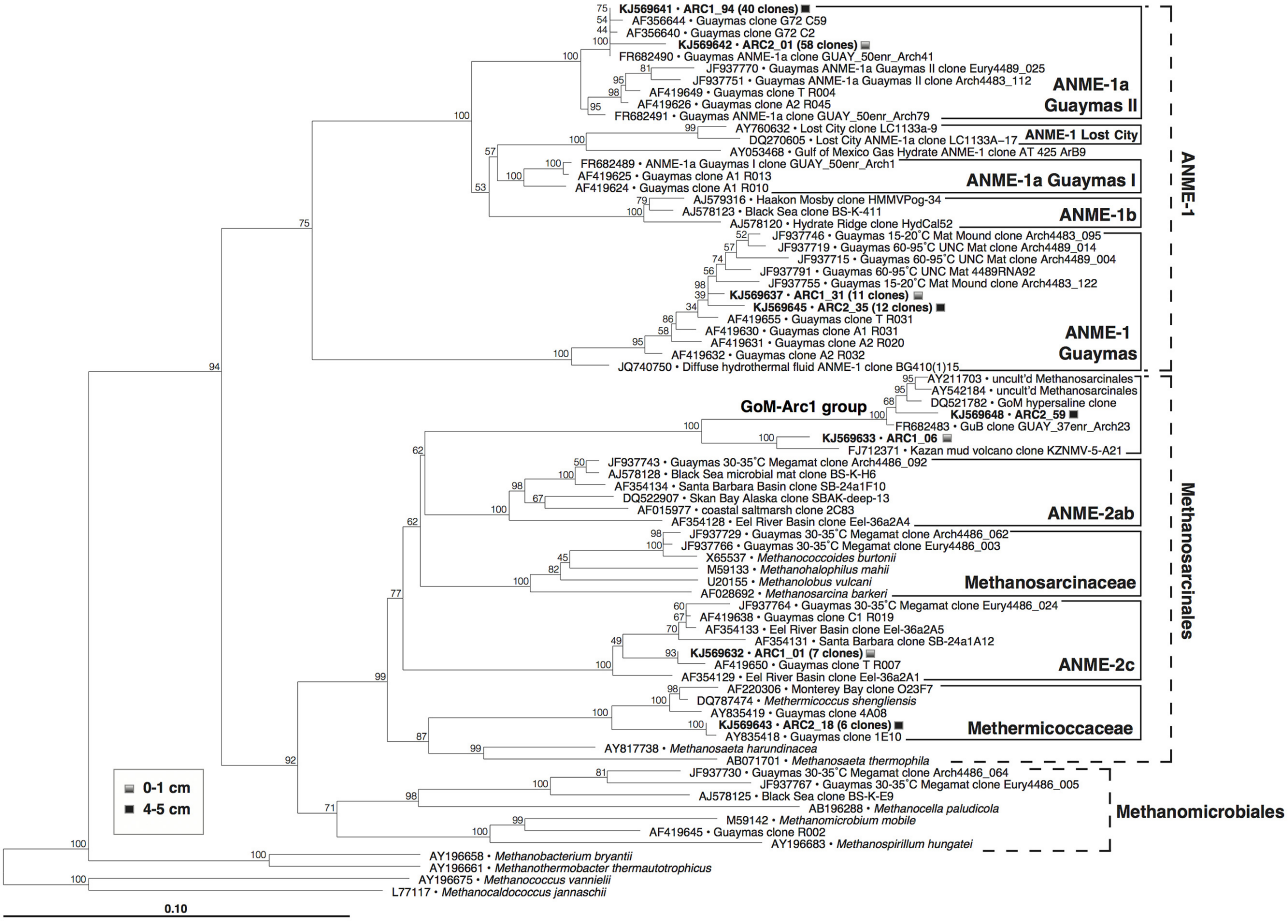
**Phylogeny of methanogens and ANME archaea in Guaymas Mat Mound hydrothermal sediments, based on partial 16S rRNA gene sequences (*E. coli* positions 28–915)**. The tree was inferred using neighbor joining based on Jukes–Cantor sequence distances, and was checked by 1000 bootstrap iterations, as implemented in ARB.

The archaeal 16S rRNA gene clone libraries from both samples (**Figure 6**) were dominated by anaerobic methane-oxidizing archaea of the ANME-1 lineage, more specifically the ANME-1a Guaymas II cluster defined previously for a subgroup of ANME-1 archaea thriving in thermophilic enrichments (Holler et al., 2011), and by members of the ANME-1 Guaymas lineage, a presumably heat-tolerant sister linage of ANME-1 that has so far only been found in Guaymas hydrothermal sediments and at diffuse vent sites (Biddle et al., 2012; Merkel et al., 2012). Smaller clone groups included ANME-2c archaea from the surface sediment, members of the *Methermicoccaceae* from the 4 to 5 cm layer, and clones within the GoM-Arc1 group (Lloyd et al., 2006) from both layers (**Figure 6**). The remaining archaeal clones were apparently unaffiliated with methane-related metabolism (Supplementary Figure S4), and were linked with uncultured, most likely heterotrophic archaeal lineages that are commonly reported from cold seafloor sediments (Marine Benthic Groups B and D, Vetriani et al., 1999; Miscellaneous Euryarchaeotal Group or MCG, Inagaki et al., 2003; for genome studies, see Lloyd et al., 2013), or the uncultured Guaymas Euryarchaeotal Group (Dhillon et al., 2005).

Consistent with the sulfate-and methane-rich geochemical regime, the surficial hydrothermal sediments (upper 5 cm) at the base of Mat Mound were conspicuously dominated by ANME-1 archaea. Both ANME-1 and ANME-1Guaymas phylotypes were detected with similar frequency in the cool surface layer and in the high-temperature sediments at 4–5 cm depth, suggesting that both groups can tolerate medium to high temperatures.

These archaeal groups were previously found to co-occur at different locations of Guaymas Basin, sampled between May 1998 and December 2009 (**Table 1**). They were initially detected in 16S rRNA gene clone libraries of surficial mat-covered sediments (2–74°C within the upper 5 cm) collected during *Alvin* Dive 3202 (Teske et al., 2002). Subsequently, the same groups were found in 16S rRNA and *mcrA* gene and transcript surveys of hot sediments covered with microbial mats (60–96°C at 10–12 cm depth, core 4489-10), sampled during *Alvin* dive 4489 (Biddle et al., 2012). They were also detected in 16S rRNA and *mcrA* gene and transcript surveys of relatively cool sediments (15–20°C at 8–10 cm, core 4483-23; Biddle et al., 2012) from the base of Mat Mound, sampled during *Alvin* dive 4483 (Supplementary Figure S1). Most recently, they were found in 16S rRNA gene clone libraries from several hydrothermal mat-covered sediments collected during *Alvin* dives 4569 and 4571 (summarized in **Table 1**; McKay et al., 2015). Consistent with these environmental distributions of phylotypes, incubation and enrichment experiments at elevated temperatures have constantly yielded types of ANME-1 archaea, but not ANME-2 or ANME-3. Mixed *mcrA* phylotypes of the ANME-1 and mcrA-Guaymas groups (the presumable *mcrA* equivalent of ANME-1Guaymas; Biddle et al., 2012) were also recovered from two *in situ* colonization devices implanted into mat-covered sediments at Mat Mound over 8 and 22 days in June 2010; the colonization chambers were exposed to mean *in situ* temperatures near 44°C and a total temperature range of 36–57°C (Callac et al., 2013). Members of the ANME-1 archaea have been enriched in laboratory incubations at 37°C (Kellermann et al., 2012), at 50°C (Holler et al., 2011; GenBank numbers FR682491 and FR682489, **Figure 6**) and at 52°C (Wankel et al., 2012). These environmental occurrence and enrichment patterns suggest that members of the ANME-1 and ANME-1Guaymas lineages tolerate wide temperature ranges. The results are congruent with a global high-throughout sequencing-based biogeographical analysis of methane seep sediments, where ANME-1 phylotypes were found to correlate with hot or subsurface seep sediments (Ruff et al., 2015), indicating that these archaea can cope with extreme conditions and populate niches that are not occupied by other ANME clades.

**Table 1:** Comparison of ANME, SEEP-SRB2, and HotSeep-1 habitats and communities in Guaymas Basin sediments.

Location	Alvin dive; core #, sediment depth	CH_4_/SO_4_/ H_2_S concentrations [mM]	Microbial mat	Approximate interpolated *in situ* temperature regime	Alvin dive and core number for closest molecular sequencing core	Clone numbers for ANME, HotSeep-1 and SEEP-SRB2 groups	Reference
Everest Mound	3203 core A 0–2.5 cm	Gas bubbles below 10 cm	Orange mat	74°C at 5 cm	same core subsample	ANME-1: 29 clones ANME-1Guaymas: 24 clones HotSeep-1: 9 clones SEEP-SRB2: 2 clones	Teske et al., 2002
Orpheus site	3207 core C 0–2.5 cm	Gas bubbles below 15 cm	bare	57°C at 5 cm	same core subsample	ANME-2c: 52 clones ANME-1: 1 clone	Teske et al., 2002
Mat Mound	4483-23 8–10 cm	3.34/9.38/1.66	White mat	15–20°C	4483-21 8–10 cm	ANME-1Guaymas: 22 clones ANME-1: 1 clone No bacterial 16S clone library available	Biddle et al., 2012
Megamat	4486-24 6–8 cm	1.05/24.93/0.006	bare	30–35°C	4486-22 6–8 cm	ANME-2c: 7 clones ANME-2ab: 1 clone No bacterial 16S clone library available	Biddle et al., 2012
UNC Mat	4489-11 10–12 cm	3.06/25.71/1.86	Orange mat	60–96°C	4489-10 10–12 cm (also 8–10 cm and 12–14 cm)	ANME-1Guaymas: 24 clones ^∗∗∗^ ANME-1: 2 clones No bacterial 16S clone library available	Biddle et al., 2012
Mat Mound	4484-3 0–1 cm	^∗^2.21/19.93/0.06	White mat	20–25°C	4484-1 0–1 cm	ANME-1: 58 clones ANME-1Guaymas: 11 clones ANME-2c: 7 clones HotSeep-1: 7 clones SEEP-SRB2: 7 clones	This study
Mat Mound	4484-3 4–5 cm	^∗∗^3.40/4.34/0.63	White mat	65–75°C	4484-1 4–5 cm	ANME-1: 40 clones ANME-1Guaymas: 12 clones HotSeep-1: 4 clones SEEP-SRB2: 6 clones	This study
Marker 14 mat	4569-9 0–3 cm	0.9/22.67 /0.88	Orange mat	21°C	same core subsample	ANME-1: 40 clones ANME-1Guaymas: 9 clones HotSeep-1: 17 clones SEEP-SRB2: 1 clone	McKay et al., 2015
Marker 14 mat	4569-9 9–12 cm	1.8/23.66 /1.91	Orange mat	49°C	same core subsample	ANME-1: 21 clones ANME-1Guaymas: 16 clones HotSeep-1: 3 clones SEEP-SRB2: 4 clones	McKay et al., 2015
Marker 14 mat	4569-9 30–33 cm	2.3/23.21/no data	Orange mat	84°C	same core subsample	ANME-1: 2 clones ANME-1Guaymas: 20 clones No bacterial 16S clone library available	McKay et al., 2015
Marker 14 mat	4569-2 0–3 cm	2.5/21.75/ 0.48	White mat	6°C	same core subsample	ANME-2c: 4 clones	McKay et al., 2015
Marker 14 mat	4569-2 24–27 cm	2.2/20.94/ 2.70	White mat	46°C	same core subsample	ANME-1: 21 clones HotSeep-1: 7 clones	McKay et al., 2015
Marker 14 mat	4569-4 0–3 cm	0.0/24.25/ 0.06	Outside of mat	4°C	same core subsample	ANME-1: 2 clones ANME-2c: 10 clones SEEP-SRB2: 1 clone	McKay et al., 2015
Marker 14 mat	4569-4 36–39 cm	0.6/ ^∗∗∗∗^26.01/ ^∗∗∗∗^0.54	Outside of mat	22°C	same core subsample	ANME-1: 1 clone ANME-2c: 12 clones	McKay et al., 2015
Marker 3 mat	4571-4 0–3 cm	2.6/21.85/ 2.16	White mat	10°C	same core subsample	ANME-1: 26 clones ANME-1Guaymas: 1 clone ANME-2c: 5 clones HotSeep-1: 2 clones SEEP-SRB2: 1 clone	McKay et al., 2015
Marker 3 mat	4571-4 36–39 cm	1.6/5.96/ 3.19	White mat	61°C	same core subsample	ANME-1: 12 clones No bacterial 16S clone library available	McKay et al., 2015

*Samples 4483-21, 4486-22, 4489-10, 4484-1 (0–1 cm), and 4484-1 (4–5 cm) correspond to samples GB1, GB2, GB3, and GB4a and GB4b in a global pyrosequencing comparison of methane seep communities (Ruff et al., 2015)*.

*^∗^Data for 1 cm; the sulfide concentration is the average of 6 cores around Mat Mound*.

*^∗∗^Data for 5 cm; the sulfide concentration is the average of 6 cores around Mat Mound*.

*^∗∗∗^Clones from DNA and RNA-based libraries*.

*^∗∗∗∗^ indicates for 33–36 cm*.

Wide temperature tolerance represents a suitable adaptation to hydrothermal pumping and episodic mixing of porewater fluids, combined with seawater entrainment (Gundersen et al., 1992). These mixing processes could also prevent the establishment of spatially separated populations over small scales; this possibility is supported by recent observations of high microbial population connectivity in surficial Guaymas Basin hydrothermal sediments (Meyer et al., 2013), and also by observations of extremely soft sediment consistency during coring and core retrieval at the base of Mat Mound. Physical mixing and wide thermal tolerances of many Guaymas microorganisms could represent the mutually supporting key factors for defining the niche and assembling the community (see Ruff et al., in review, for a contrasting low-connectivity example). The working hypothesis of high connectivity does not explain all features of the dataset, such as the detection of ANME-2c archaea only in the clone libraries of the sediment surface layer, or the lack of *Archaeoglobales* clones which were abundant in the previously analyzed core 4483-23, collected nearby at the base of Mat Mound (Biddle et al., 2012). 16S rRNA gene clone library results remain imperfect indicators for the presence or absence of a microbial group, as demonstrated by previous comparative 16S rRNA, functional gene and V6-tag analyses in Guaymas Basin (Biddle et al., 2012).

We propose that the geochemical regimes in Guaymas Basin sediments are linked to distinct ANME populations: Highly reducing, methane-rich, high-flow, and high-temperature conditions select for ANME-1/ANME-1Guaymas, whereas moderately reducing, lower-flow and moderate temperature conditions appear to be tolerated by ANME-2 but not ANME-1. The occurrence range of ANME-2c extends into sediments with lower sulfide concentrations and no microbial mat cover, consistent with lower hydrothermal flow (**Table 1**); this pattern observed here is congruent with previous reports that assign this clade to low-fluid flow seeps characterized by increased bioturbation and oxygen exposure (Knittel et al., 2005; Felden et al., 2014). Potentially divergent habitat and temperature preferences for ANME-1 and ANME-1Guaymas archaea in the Mat Mound sediments are harder to resolve, since the environmental controls that are regulating the distribution patterns of these lineages remain open to ambiguities, for example the possibility of fluctuating temperature regimes *in situ* (McKay et al., 2015) and high population connectivity within shallow sediments (Meyer et al., 2013). Yet, the persistent detection of ANME-1Guaymas at hot *in situ* temperature regimes, including 65–75°C (core 4484-1; this study), 60–96°C (core 4489-10; Biddle et al., 2012), and up to 84°C (core 4569-9; McKay et al., 2015), together with the conspicuously GC-enriched 16S rRNA gene of this ANME group (Merkel et al., 2012), suggest that the ANME-1Guaymas lineage has a consistently high temperature tolerance. The ANME-1Guaymas group has not been detected outside of Guaymas Basin hydrothermal sediments (Biddle et al., 2012) or diffuse venting locations at mid-ocean ridges (Merkel et al., 2012), and is therefore a strong candidate for a high-temperature-tolerant ANME lineage specialized for hydrothermal environments.

### Diversity and Function of Bacterial Groups

**FIGURE 7:**
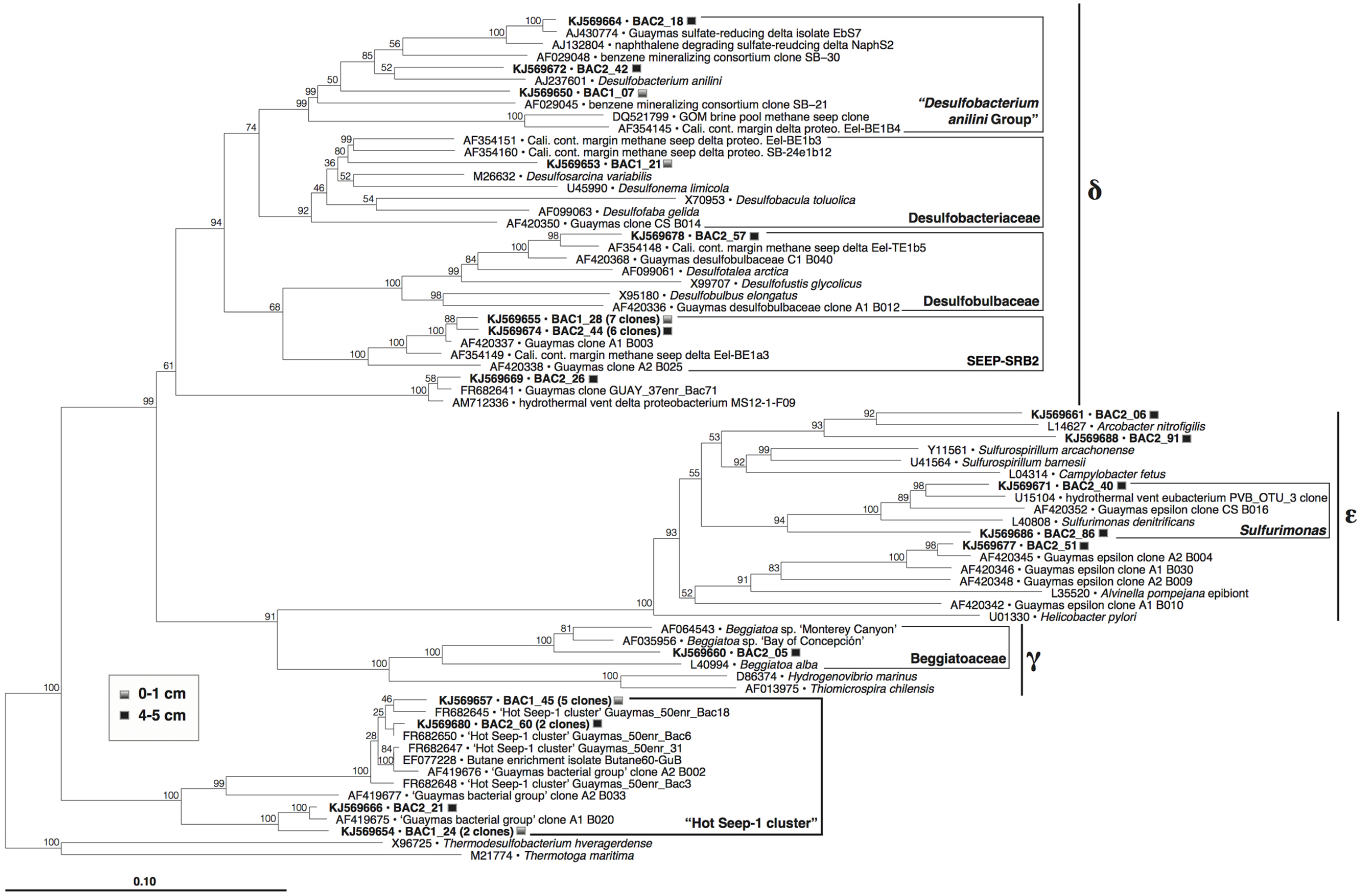
**Phylogeny of *Proteobacteria* and the HotSeep-1 Group in Guaymas Mat Mound hydrothermal sediments, based on near-complete 16S rRNA gene sequences (*E. coli* positions 28–1491)**. The tree was inferred using neighbor joining based on Jukes–Cantor sequence distances, and was checked by 1000 bootstrap iterations, as implemented in ARB.

Within the complex bacterial community, the largest clone groups consist of members of sulfate-reducing and sulfur-oxidizing lineages within the *Proteobacteria* (**Figure 7**). Epsilonproteobacterial clones related to the sulfur-oxidizing genera *Arcobacter* and *Sulfurimonas*, and a clone within the nitrate-reducing, sulfur-oxidizing *Beggiatoaceae* (Candidatus Group Maribeggiatoa, Teske and Salman, 2014) indicate the presence of sulfur-oxidizing bacterial populations. Other bacterial clones included six within the *Chloroflexi*, four within the *Caldithrix* lineage, and one to two clones each within fourteen other deeply branching, phylogenetic lineages (Supplementary Figure S5), including those that contain cultured anaerobic thermophiles (*Deferribacteres*, *Caldithrix*, *Thermotoga*, *Caldiserica*/OP5).

Sulfate-reducing family-level lineages within the *Deltaproteobacteria* include the “*Desulfobacterium anilini* group” of aromatic hydrocarbon-degrading specialists (reviewed in Teske, 2010), *Desulfobacteraceae*, *Desulfobulbaceae*, and the SEEP-SRB2 lineage. The SEEP-SRB2 lineage is of considerable interest as a hydrocarbon seep specialist; this group was originally detected in methane seep sediments of the Eel River Basin offshore California (Orphan et al., 2001), and subsequently in diverse seep sediments (Knittel et al., 2003), often underneath sulfur-oxidizing microbial mats (Lloyd et al., 2010; Kleindienst et al., 2012; Vigneron et al., 2014) including Guaymas Basin (Teske et al., 2002); it has also been enriched in long-term high-pressure methane-oxidizing incubations (Timmers et al., 2014). SEEP-SRB2 occurs often as single cells or monospecific aggregates, although they are also capable of forming consortia, clusters and homogeneous mixtures with ANME-2c cells, and with ANME-1 cells and filaments (Kleindienst et al., 2012; Yanagawa et al., 2013; Ruff et al., in review). The SEEP-SRB2 bacteria were proposed as candidates for the oxidation of short-chain alkanes (Kleindienst et al., 2012), which would be consistent with their detection in the short-chain alkane-rich Mat Mound sediments in this study. Detecting the group in hydrothermal sediments (this study, and Kleindienst et al., 2012) could indicate some degree of tolerance for elevated temperatures, although it must be noted that most observations of SEEP-SRB2 come so far from cold or mesophilic sediments and enrichments. Its closest cultured sister lineage is currently represented by the thermophilic sulfur-disproportionating bacterium *Dissulfuribacter thermophilus* (Slobodkin et al., 2013).

Several clones fell into the HotSeep-1 group (**Figure 7**), a bacterial lineage that was alternately reported as an affiliate of the *Deltaproteobacteria* (Holler et al., 2011; Adams et al., 2013) or as an independently branching bacterial lineage (Teske et al., 2002), as consistent with our phylogeny (Supplementary Figure S5). In contrast to SEEP-SRB2, HotSeep-1 occurs consistently in sediments with high temperatures, as shown in a global pyrosequencing survey of methane seep sediments (Ruff et al., 2015). HotSeep-1 cells were detected by CARD-FISH hybridization in syntrophic association with ANME-1 archaea in methane-oxidizing enrichments at 50°C, inoculated with Guaymas Basin sediments (Holler et al., 2011). Enrichment and culture studies with HotSeep-1 bacteria from Guaymas Basin sediments showed that these organisms can grow independently as thermophilic hydrogenotrophs, but they also form thermophilic methane-oxidizing consortia with ANME-1 archaea; under the latter conditions they reduce their hydrogenotrophic activity in favor of direct electron transfer from their ANME-1 syntrophs through nanowire-like cell-to-cell connections (Wegener et al., 2015).

The HotSeep-1 group was also frequently detected in thermophilic sulfate-reducing enrichment cultures on *n*-butane from Guaymas Basin at 60°C (Kniemeyer et al., 2007), and in sulfate-reducing enrichments on ethane, propane, and *n*-butane at 55°C inoculated with alkane-rich sediments from the Middle Valley hydrothermal field (Adams et al., 2013). As a caveat, these enrichments did not proceed to the pure culture stage, in contrast to other sulfate-reducing short-chain alkane oxidizers (Kniemeyer et al., 2007); it is therefore likely that the members of the HotSeep-1 group do not oxidize ethane, propane or *n*-butane directly but play an indirect role, for example as in syntrophic alkane degradation (Zengler et al., 1999). Thus, HotSeep-1 bacteria could be viewed as versatile syntrophs that play the role of electron/hydrogen sink within different consortia, in the thermophilic temperature range of 50–60°C.

## Conclusion and Outlook

The Mat Mound dataset highlights characteristic features of the surficial hydrothermal sediment environment of Guaymas Basin: coexistence of high concentrations sulfate and methane in the porewater; availability of short-chain alkanes of thermogenic origin; conspicuous microbial mats of sulfur-oxidizing bacteria on the sediment surface; highly reduced conditions in conjunction with steep temperature gradients; and frequent detection of ANME-1/ANME-1Guaymas archaea and of the HotSeep-1 and SEEP-SRB2 bacteria in the sediments (**Table 1**). Their co-occurrence in these hydrothermally active sediments indicates similar preferences for hydrothermal substrates, and either tolerance or preference for the elevated temperatures that characterize these sediments. As a working hypothesis, this mixed bacterial and archaeal community catalyzes the oxidation of both methane and short-chain alkanes, and constitutes a microbial community signature that is characteristic for hydrothermal or cold seep sediments containing both substrates. While *in situ* methane and alkane availability and strongly reducing conditions constitute the primary chemical control on these communities, temperature would superimpose an additional layer of control that selects for specifically thermophilic community members, as previously suggested (Biddle et al., 2012; Ruff et al., 2015). Specific predictions could be tested by detailed biogeographic studies of hydrocarbon-rich seeps and vents that link different temperature regimes with fine-scale (genus-level) phylogenetic resolution; such a strategy would be necessary for linking ANME subclades to their specific habitat preferences, or for tracking the HotSeep-1 and SEEP-SRB2 groups. At last, such inferences can be checked and complemented by pure-culture studies and completed genomes of microbial key players.

## Author Contributions

FD performed the clone library and sequence analysis and wrote the first manuscript draft, supported by ZC and SD. MK and JL joined the Guaymas Basin research cruise and performed, plotted and commented on the short-chain alkane and hydrogen measurements in the lab of KH who was leading these aspects of the study. SR re-analyzed and plotted the V6-tag data. JB performed the initial V6-tag analyses, and together with LM, BM, KL, DA, and HM joined the Guaymas Basin research cruise and performed shipboard sample processing and porewater geochemical measurements. AT headed the cruise, designed the study and wrote the manuscript with input from all coauthors.

## Conflict of Interest Statement

The authors declare that the research was conducted in the absence of any commercial or financial relationships that could be construed as a potential conflict of interest.

## Footnotes

1 http://www.ncbi.nlm.nih.gov/BLAST/.

2 www.arb-home.de.

3 http://vamps.mbl.edu/resources/prim.php.
