# Supplementary material for: Microbial Communities in Methane- and Short Chain Alkane-Rich Hydrothermal Sediments of Guaymas Basin
Source: Front Microbiol. 2016 Jan 29;7:17. doi: 10.3389/fmicb.2016.00017 (PMC4731509; doi:10.3389/fmicb.2016.00017)

**Supplementary Online Material**

**
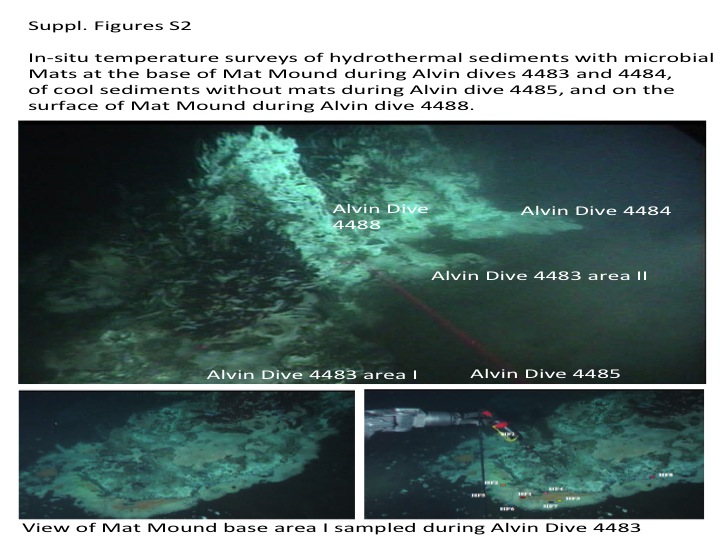
Figure S1.** Compilation of *in-situ* temperature and coring surveys of hydrothermal sediments with microbial mats at the base of Mat Mound during Alvin dive 4483 (areas I and II), of cool sediments without mats during Alvin dive 4485, and on the surface of Mat Mound during Alvin dive 4488. The temperature plots show temperature in °C on the x-axis and sediment depth in centimeters on the y-axis. Microbial mat and sediment sampling sites were annotated with temperature and coring locations, as recorded *in-situ* and recovered post-dive using the Alvin framegrabber tool (http://4dgeo.whoi.edu/alvin ).


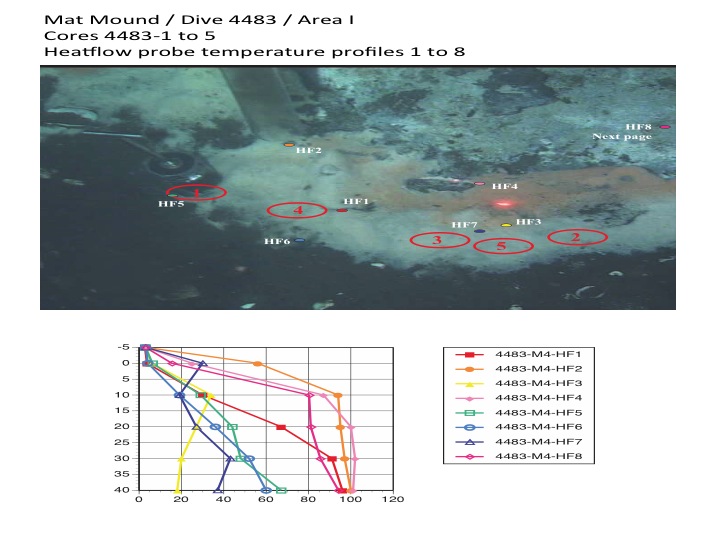


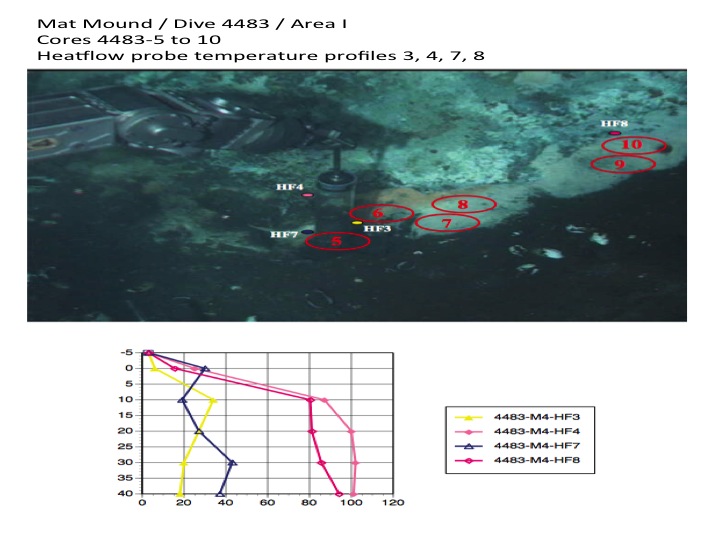


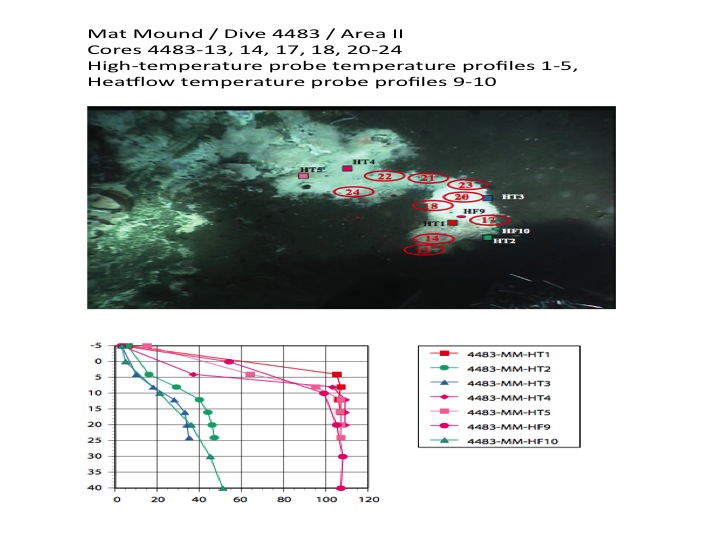


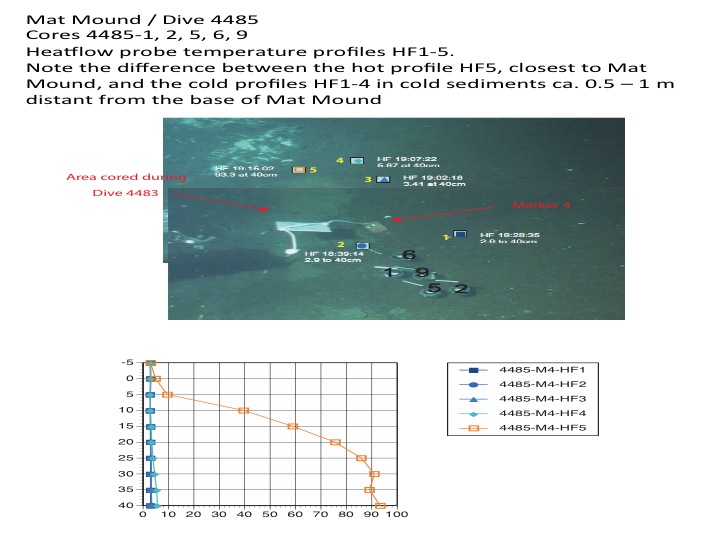


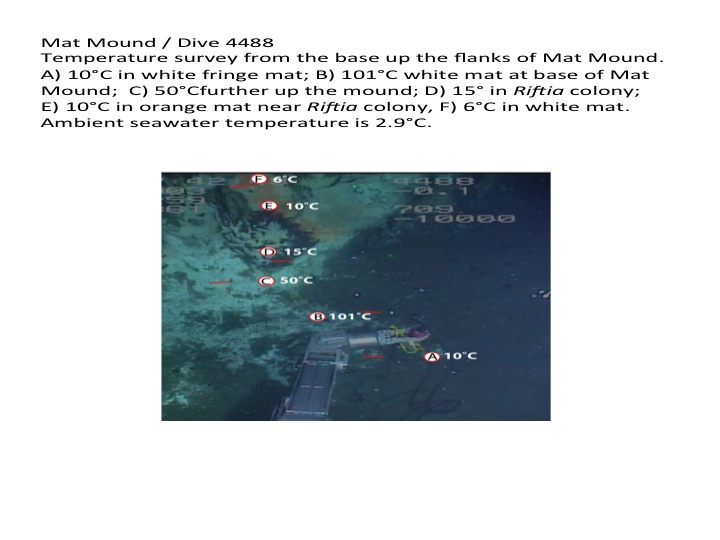


**Figure S2.** Compilation of *in-situ* temperature and coring surveys of hydrothermal sediments with microbial mats at the base of Mat Mound during Alvin dive 4484, including the cores 4484-1 and 4483-1 used in this study. A) Photographs of *in-situ* temperature measurements using the Heatflow (HF) probe in the center of the white mat, where HF profile 3 was measured (left and center) and towards the edge of the mat, where the cooler HF profile 4 was measured (right). B) Photographs of *in-situ* temperature measurements using the High-temperature probe (HT) of Alvin, measuring a hot HT profile (HT1) between HF profiles 3 and 4, and a cool HT profile (HT2) at the edge of mat, outward from HF4. C) Sampling grid for temperature profiles and core locations at the Mat Mound mat during Alvin Dive 4484. Images of the microbial mat and sediment sampling sites were annotated with temperature and coring locations, as recorded *in-situ* and recovered post-dive using the Alvin framegrabber tool (http://4dgeo.whoi.edu/alvin).

**
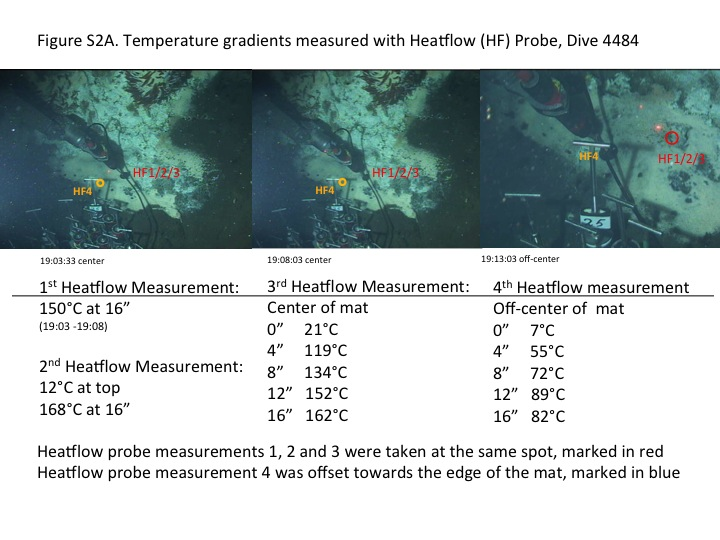
**

**
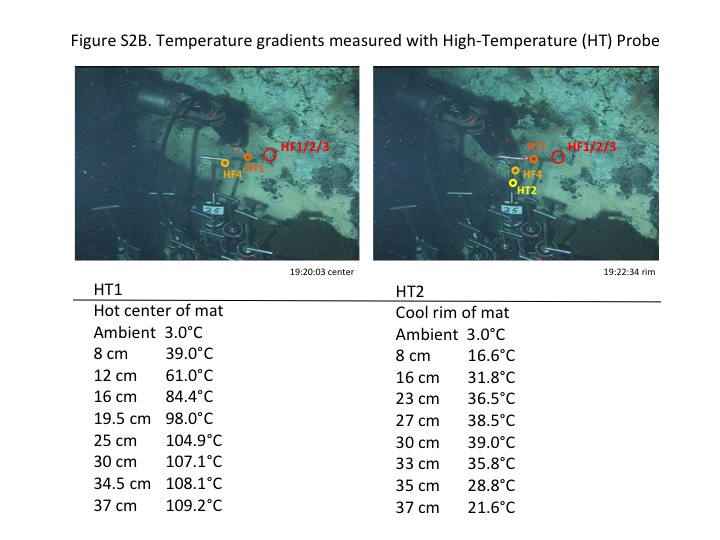
**

Figure S2C. Sampling map with core positions and plotted temperature gradients

**
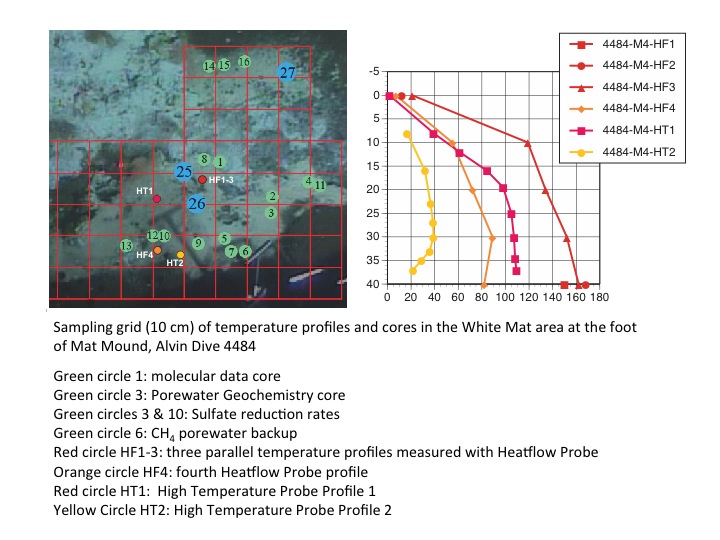
**

**Figure S3**. Sulfate reduction rates measured *ex-situ* at 20°C in 2 cm intervals for sediment cores 4483-3 and 4483-10. Note the different vertical extent of the y-axis (12 cm in 4483-3, 16 cm in 4483-10). The error bars are derived from triplicate measurements, when available.

**Figure S4.** Phylogeny of uncultured archaeal lineages in Guaymas Mat Mound hydrothermal sediments, based on partial 16S rRNA gene sequences (*E. coli* positions 28 to 915). The tree was inferred using neighbor joining based on Jukes-Cantor sequence distances, and was checked by 1000 bootstrap iterations, as implemented in ARB.


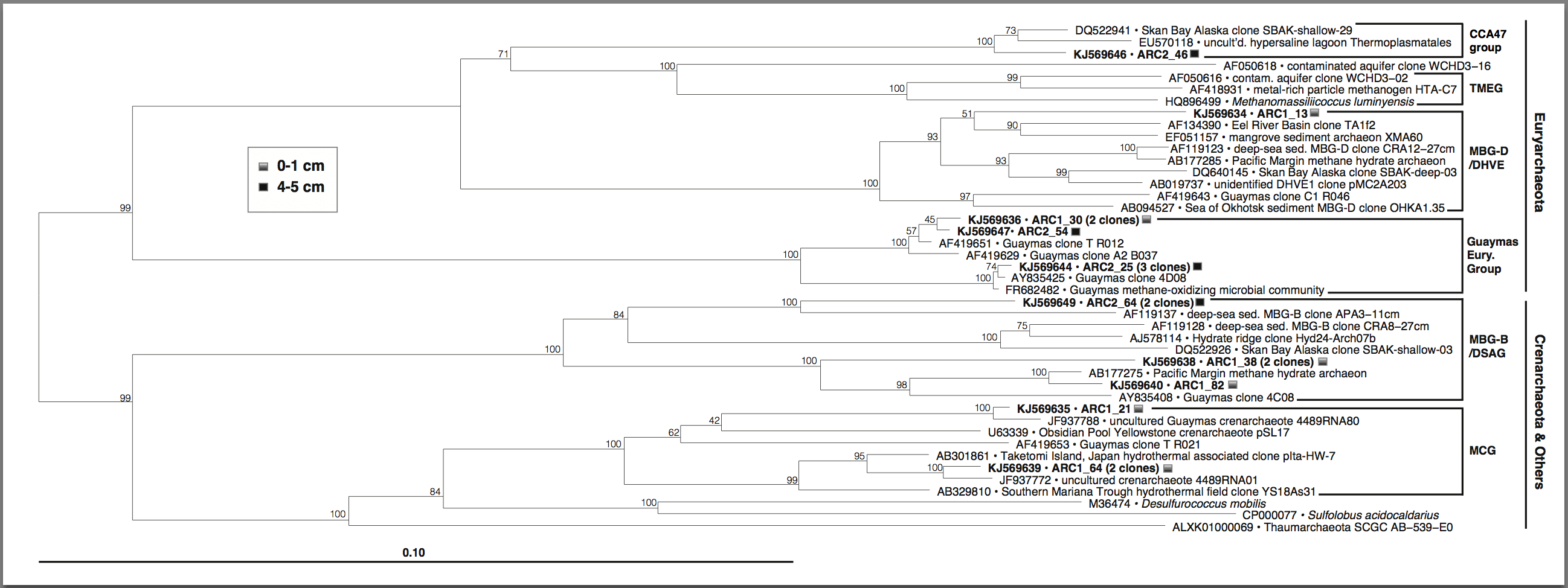


**Figure S5.** Phylogeny of bacterial lineages in Guaymas Mat Mound hydrothermal sediments, based on near-complete 16S rRNA gene sequences (*E. coli* positions 28 to 1491). The tree was inferred using neighbor joining based on Jukes-Cantor distances, and was checked by 1000 bootstrap iterations, as implemented in ARB.


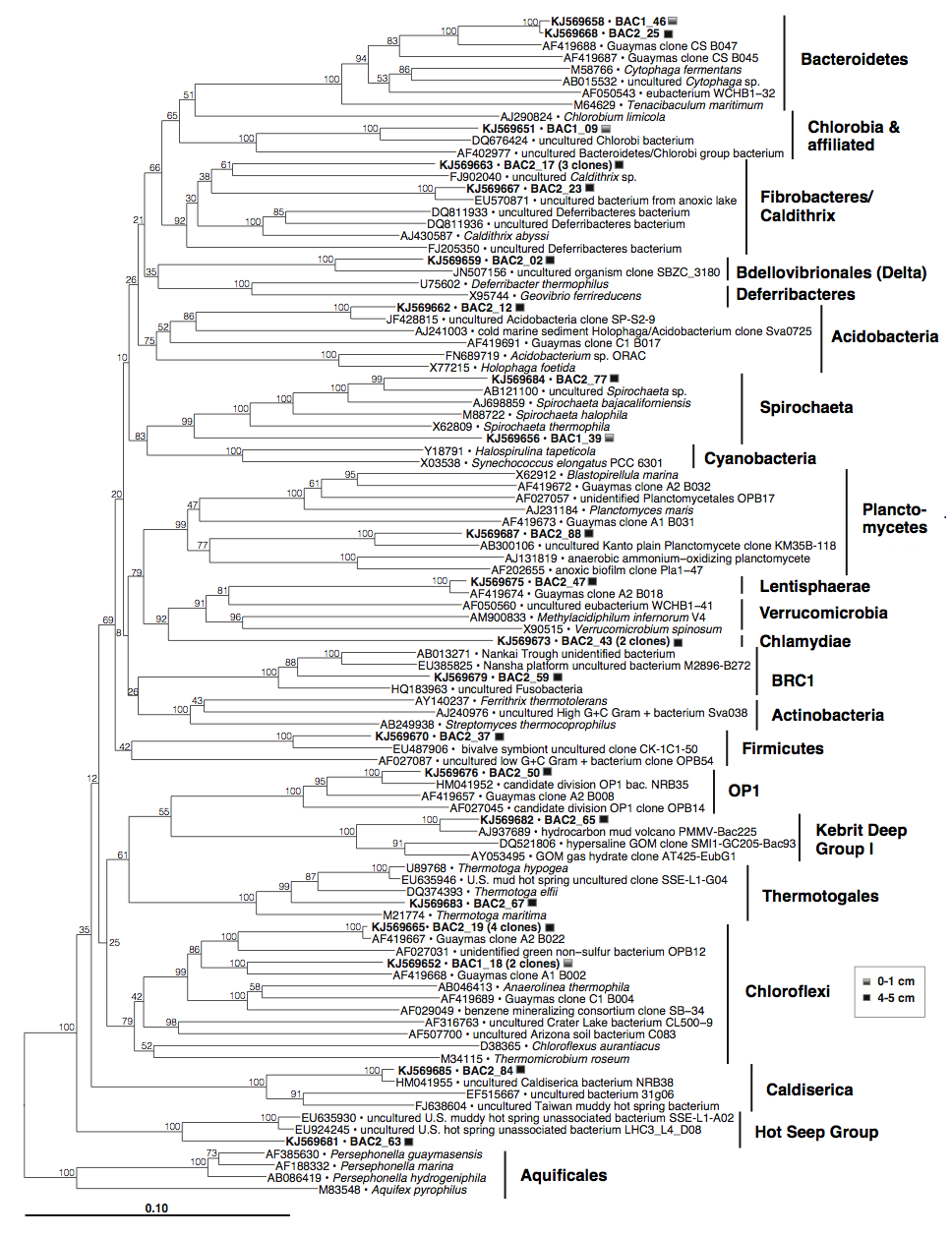


Table S1: Porewater geochemistry data in Mat Mound cores


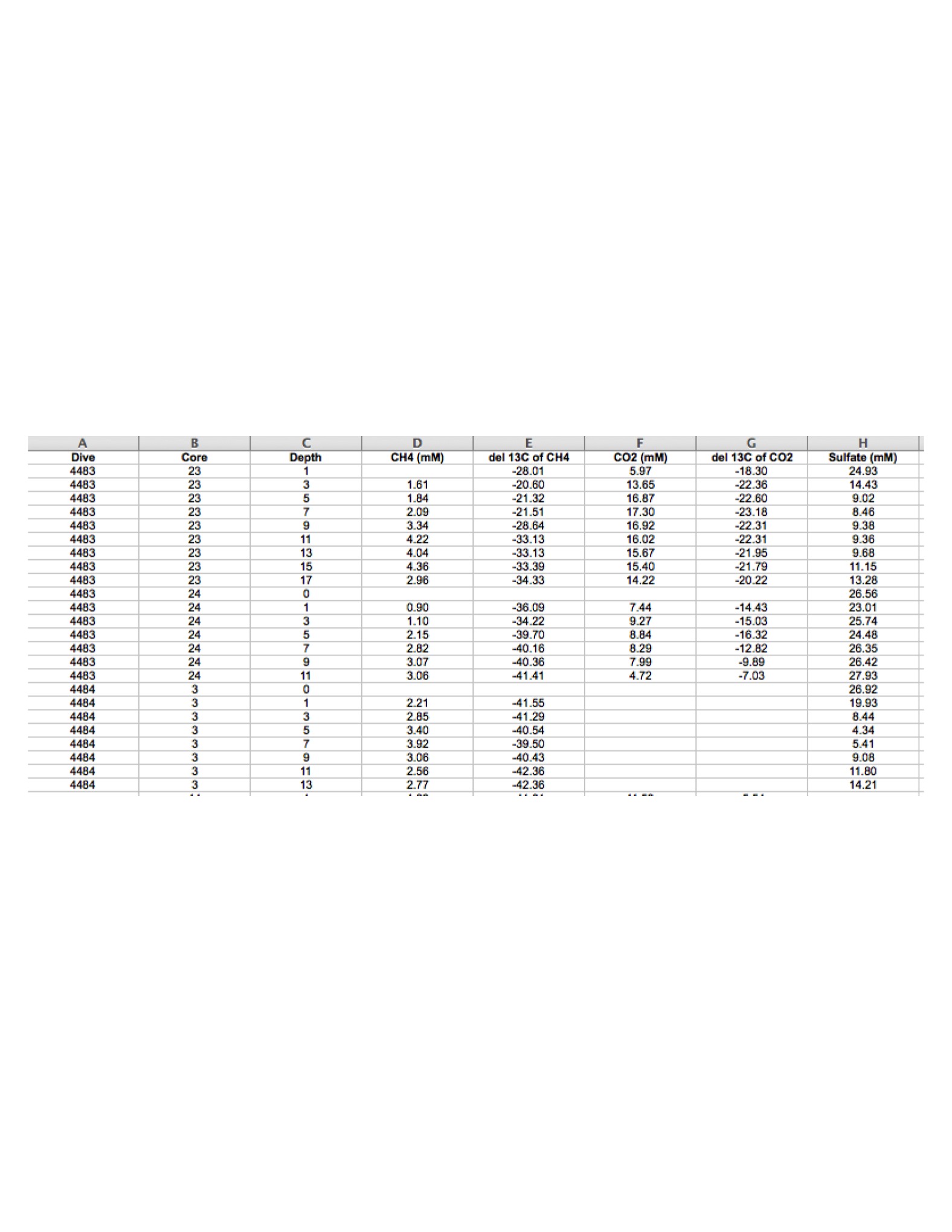


Table S2: Porewater sulfide concentrations [mM] in Mat Mound cores


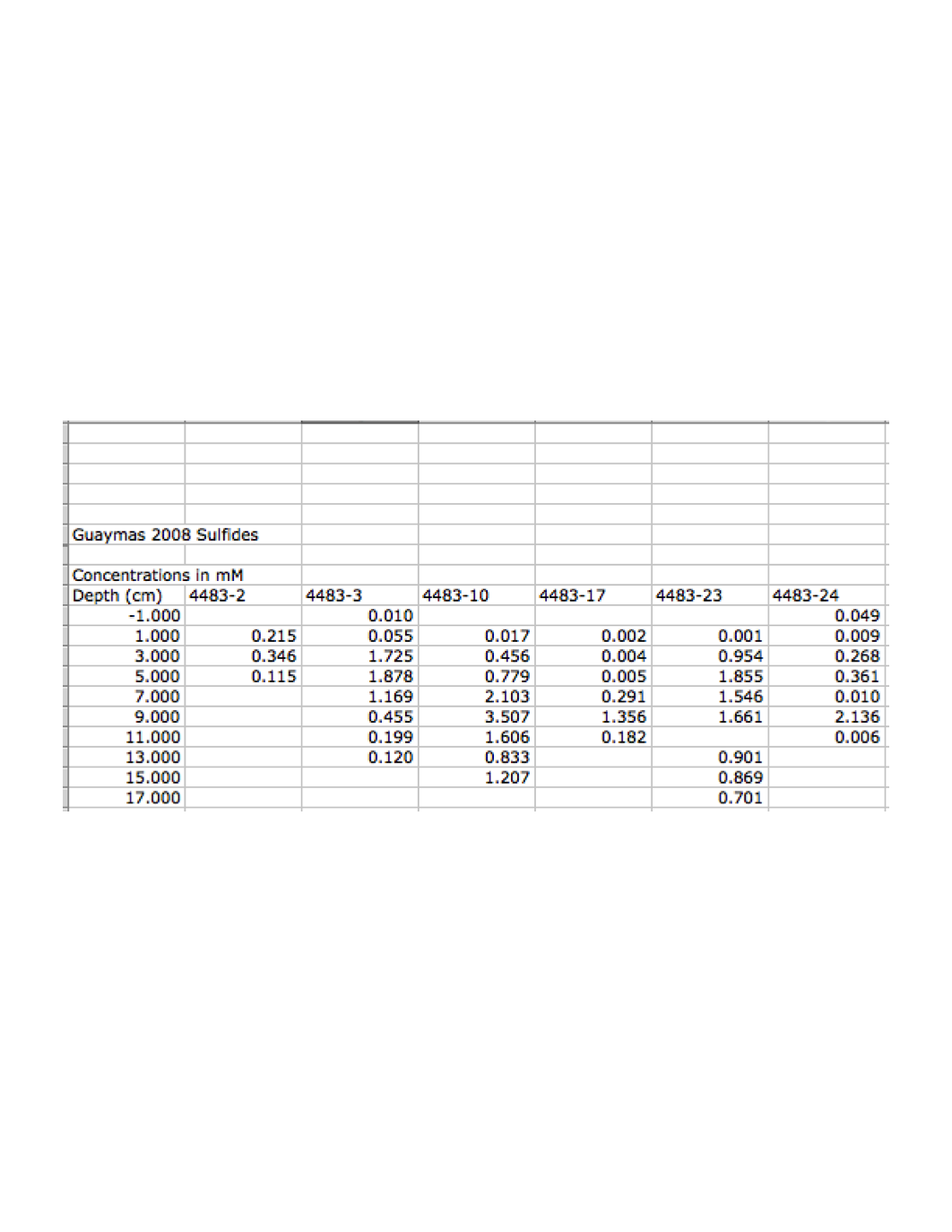

Supplement: Supplementary file 1 [file Data_Sheet_1.DOCX]
